# Cervical Cancer Screening in Refugee and Migrant Populations: Results of Systematic Review and Meta-Analysis in Cross-Sectional and Cohort Studies

**DOI:** 10.3390/cancers17182966

**Published:** 2025-09-10

**Authors:** Vincenzo Restivo, Davide Graci, Angelo Immordino, Daniele Giacomo Mancuso, Fátima Morales, Chiara Pace, Alessandra Pirrello, Alessandra Casuccio, Palmira Immordino

**Affiliations:** 1 School of Medicine, University Kore of Enna, 94100 Enna, Italy; vincenzo.restivo@unikore.it; 2 Department of Health Promotion, Mother and Child Care, Internal Medicine and Medical Specialities, University of Palermo, 90127 Palermo, Italy; davide.graci01@unipa.it (D.G.); danielegiacomo.mancuso@unipa.it (D.G.M.); chiara.pace@unipa.it (C.P.); alessandra.pirrello@unipa.it (A.P.); alessandra.casuccio@unipa.it (A.C.); 3 Otorhinolaryngology Section, Biomedicine, Neuroscience and Advanced Diagnostic Department, University of Palermo, 90127 Palermo, Italy; angelo.immordino182@gmail.com; 4 Department of Preventive Medicine and Public Health, University of Seville, 41004 Seville, Spain; fmmarin@us.es; 5 Sbarro Institute for Cancer Research and Molecular Medicine, Center for Biotechnology, College of Science and Technology, Temple University, Philadelphia, PA 19122, USA

**Keywords:** cervical cancer screening, cancer prevention, HPV, migrant health, healthcare access

## Abstract

Background/Objectives: Cervical cancer is currently the fourth leading cause of cancer in women. It is primarily caused by Human Papilloma Virus (HPV) infections. Primary prevention methods, such as vaccines, and secondary prevention strategies, such as screening, have significantly reduced the burden of these diseases. The screening could be a crucial factor in the early diagnosis. This study aims to estimate the access of migrant and refugee populations to cervical cancer screening (CCS). Methods: A meta-analysis of scientific literature present in Pubmed and Scopus databases was conducted according to the PRISMA 2020 guidelines. Eighty-seven cross-sectional and five cohort unique studies were examined, to evaluate the participation of migrant and refugee populations to CCS programs in different world regions. Results: Statistical analysis was performed using STATA 14.2 software. Among cross-sectional studies, mean regular adherence to CCS for migrant and refugees resulted being 56% (95% CI 53–60), while participation at least once is 60% (95% CI 54–65). In cohort studies, regular adherence and participation at least once are, respectively, 55% (95% CI 50–59) and 56% (95% CI 52–61). Conclusions: The results of this review show how migrant and refugee populations have limited access to prevention interventions due to several socio-cultural factors. Our work calls for public health professionals’ efforts in order to promote more inclusive policies and prevention strategies towards those populations, aiming to reduce disparities and public health expenditures.

## Simple Summary

Cervical cancer is one of the most common cancers in women, but it can often be prevented through vaccination and regular screening tests. Unfortunately, not all groups of women benefit equally from these prevention programs. Migrant and refugee women, in particular, may face cultural, financial, and social barriers that make it harder for them to access screening. In our study, we reviewed results from 92 scientific papers to understand how often women from these groups participate in screening. We found that, on average, only about half of migrant and refugee women regularly attend screening, which is much lower than expected. These findings highlight the urgent need for health systems to design prevention programs that are inclusive and culturally sensitive. By improving access for vulnerable populations, health professionals can help reduce health inequalities and prevent unnecessary illness and death from cervical cancer.

## 1. Introduction

Cervical cancer (CC) is the 4th most common cancer and the 1st cause of cancer-related mortality among women of reproductive age worldwide [1]. In 2022, there were an estimated number of over 660,000 new cases and almost 350,000 deaths, with approximately 90% of them occurring in low- and middle-income countries [2]. This cancer appears to be steadily increasing in sub-Saharan Africa [3], making its incidence in Southern Africa the highest in the world [4].

Human Papilloma Virus (HPV) is the main etiological agent responsible for CC worldwide [1]. HPV is mainly transmitted through sexual intercourse. Globally, HPV prevalence represents a worryingly high burden in pregnant women, especially in underprivileged communities and in low and middle-income countries [5]. Moreover, CC incidence is higher in rural areas than in urban ones worldwide. This could be explained due to the influence of inequity in accessibility to CC prevention measures, among others [6].

We could completely prevent CC by implementing highly effective primary and secondary prevention measures, respectively, HPV vaccination and proper screening programs. However, these measures are not equally implemented in all countries. In 2020, less than 30% of low- and middle-income countries (LMICs), while more than 80% of high-income countries (HICs) implemented them [7]. In HICs, the wide use of primary prevention has led to the enormous decrease in incidence of CC [8] and proper implementation of screening programs can further reduce it [9].

In 2018, the World Health Organization (WHO) proposed the “Cervical Cancer Elimination Initiative” to eradicate CC globally through more rigorous vaccination and screening methods [10]. The strategy defines elimination as reducing the number of new cases annually to 4 or fewer per 100 thousand women and setting three targets to be achieved by 2030: (I) 90% of girls aged 15 fully vaccinated, (II) 70% of women aged 35–45 properly CC screened, (III) 90% women with cervical disease receiving treatment [11].

The vaccination program against HPV started in 2007 [8] with Australia being the first country to introduce a government-funded HPV Vaccination Program [4]. Nowadays, 131 countries (67.52% of the WHO Member States) have fully adopted HPV vaccination. However, there is still a lack of vaccination in some of the most populous countries, such as India, China, Russia, and most of Central and Middle East Asian and African countries [12].

Although HPV vaccination has been highly effective in reducing CC, it does not protect against all HPV types, making regular CC screening an essential secondary prevention tool to detect early CC and achieve the WHO strategy by 2030 [10]. Women who have regular CC screening are 65% less likely to develop CC [13].

Current CC screening methods utilize HPV-based testing, complementing the Papanicolaou test (PAP test), developed by Dr. George Papanicolaou, a Greek physician and physiologist in 1928. The WHO recommend a PAP test every three years for women aged 21–65 and more frequently for immunocompromised ones [14]. HPV nucleic acid amplification test (NAAT) is also available for the early detection and/or screening of HPV-infected cervical cancer. In addition, visual inspection with acetic acid (VIA) [12] and HPV self-sampling test (HPV-SST) can also be used.

In HPV-SST, women receive a kit, being able to obtain their own vaginal sample. Then, they mail the sample to a laboratory to test the presence of the virus. It could be useful for populations not regularly attending PAP [15]. The WHO declared that HPV-SST could help to achieve the target of 70% of women screened using a high-performance test by the age of 35, and again by the age of 45 by 2030. This could allow women who are offered regular screening programs to get tested in their convenience of their own houses or residences [10]. Nowadays, HPV testing is employed to validate PAP results but can be used as first choice for primary CC screening, yielding higher accuracy rates than PAP test [4]. PAP test combined with HPV test are the gold standard for CC screening [13].

Despite the availability of vaccination and screening, several barriers continue to hinder widespread adoption and adherence to cervical cancer prevention measures. These challenges include low awareness and limited knowledge about CC, screening options, and HPV vaccination, particularly among women in underprivileged or rural settings [6]. Accessibility also remains a key obstacle, as remoteness and scarcity of medical facilities often prevent timely participation in screening programs [6]. In addition, decreased recommendations and outreach from healthcare professionals reduce opportunities for women to be informed and referred to preventive services [6,16]. Finally, financial constraints, including the need for out-of-pocket expenses, further limit access to both screening and vaccination, especially in populations already experiencing social and economic vulnerability [17]. Together, these factors contribute to persistent disparities in CC prevention between and within countries, emphasizing the need for targeted strategies to address these obstacles.

A study conducted in the United States shows that foreign-born women are twice as likely as native women to have never received a PAP test, underscoring how cultural barriers can hinder access to prevention services [16]. Healthcare workers must be aware of these challenges and provide targeted resources to help migrants, refugees, and asylum seekers. Moreover, un-or under-insured women-many of whom are migrants-reported lower utilization of preventive care including mammogram, PAP and HPV vaccination than US nationals [17].

These and other barriers reported in the literature highlight how the migrant and refugee population faces profound difficulties in accessing healthcare, including so-called ‘essential’ services. These challenges are related to situations of vulnerability and are often compounded by insufficient institutional support.

Successful approaches to implementing cancer-screening programs in vulnerable populations include exposure to health promotion interventions, informing individuals about cancer-screening programs, and encouraging them to seek medical attention [18]. Moreover, HPV-SST has been identified as a potential strategy to increase CC screening participation rates among migrant women [19]. Tailored screening and vaccination campaigns, specifically designed for vulnerable populations, have been suggested as methods to overcome barriers and reduce CC and HPV infections in these groups [6].

The objective of this meta-analysis is to evaluate global adherence to cervical cancer screening among migrant and refugee populations.

## 2. Materials and Methods

### 2.1. Search Strategy and Selection Criteria

A systematic literature review was conducted following the Preferred Reporting Items for Systematic Review and Meta-Analysis (PRISMA) Statement 2020 [20] guidelines. The systematic review protocol was registered on the International Prospective Register of Systematic Reviews (PROSPERO—reference number CRD42024501796). The studies were evaluated based on the PICO (Population, Intervention, Comparison, Outcome) framework. The study population included international migrants (including asylum seekers, regular migrants, migrants in irregular situations, labor migrants) and refugees, defined according to the definition of the United Nations Refugee Convention [21]. The intervention of interest was screening for the prevention of cervical cancer (CCS), as a secondary prevention strategy. Specifically, the review sought to identify factors that could increase acceptance and adherence to screening among migrant and refugee populations, considered as vulnerable groups. The comparison was made with populations that had not undergone screening, focusing on those factors that either hindered or facilitated compliance. The primary outcome assessed was the participation rates in cervical cancer screening. The initial research was conducted on Pubmed and Scopus databases, looking for scientific articles up to 31 March 2024, using the search string: “((transients and migrants) OR (migrants) OR (refugees) OR (nomads)) AND ((cervical neoplasia) OR (cervical cancer) OR (HPV) OR (papillomavirus))”.

The inclusion criteria for evaluating these studies were:

- Peer-reviewed primary studies in English, designed as cohort or Cross-Sectional;
- Studies reporting outcome measures related to cervical cancer prevention strategies, especially cervical cancer screening;
- Studies that investigated the aforementioned outcomes in populations with migratory backgrounds.

The exclusion criteria were:

- Articles not in English;
- Articles without abstracts;
- Articles whose study design was one of meta-analysis, trial, review or systematic review, pre-post study, articles appearing as opinions, guidelines, books, commentaries;
- Articles that did not include our reference population;
- Articles that did not provide outcomes related to CCS.

### 2.2. Report Evaluation and Data Extraction

The evaluation process of the studies included an initial selection phase during which duplicates were first removed (IdentificationPhase). Subsequently, the reviewers were divided into pairs (four pairs in total) to perform the screening. This step included only the publications which, according to the title and abstract, met the inclusion criteria. The pairs worked independently to increase the reliability of the work performed (Screening Phase). In the following phase, the four pairs of reviewers, again working independently, assessed the full texts (Quality Assessment phase) to decide on their inclusion or exclusion. During the Screening and Quality Assessment Phases, an additional reviewer contributed to the overall evaluation of the results and intervened in case of disagreement between the reviewers.

Data extraction was performed using a MS Excel-based database. For each report were identified:

- Migrant status of the study population, classified as “International Migrants”, “Refugees”, or “Mixed” (when methodological constraints prevented clear distinction).
- The geographical region in which the study was conducted, according to the WHO regional offices grouping [22].
- The economic level of the country of origin, based on the World Bank classification [23]. For studies where the population’s origin was unknown or multiple, the income level was coded as “unspecified”.

The main variables investigated in the study pertained to health data relating to CCS. These included whether individuals had undergone screening at least once in their lives, whether they were compliant with the host country’s recommendations on CCS, and whether the test was offered free of charge. In our review, for studies that reported only regular adherence, this information was considered for both the variables: adherence and participation at least once.

Further elements investigated concerned the socio-demographic characteristics of the study population, specifically: age, nationality, religion, marital status, number of children, educational level, knowledge of the host country’s language, family income, and time since arrival in the host country. Furthermore, we identified barriers and facilitators to screening, categorized them, and collected data to carry out a comprehensive analysis. After completing the data extraction, the quality of cohort studies was assessed using the Newcastle-Ottawa Scale (NOS) [24]. An adapted version of the NOS was used for cross-sectional studies.

### 2.3. Data Analysis

A random effects model meta-analysis of proportions was conducted to estimate the overall adherence of migrant populations to CCS worldwide. Data analysis was performed using STATA version 14.2. Furthermore, CCS participation was also stratified by population category, region of residence and income of the country of origin. If quantitative data were available for more than one study, studies with different designs were analyzed separately. Due to the extreme variability in methods used to collect socio-demographic information across studies, correlation analyses could not be performed.

The heterogeneity between studies was evaluated using the I^2^ statistic, with values > 75% considered indicative of high heterogeneity. Publication bias was evaluated separately for different study designs.

## 3. Results

**Figure 1:**
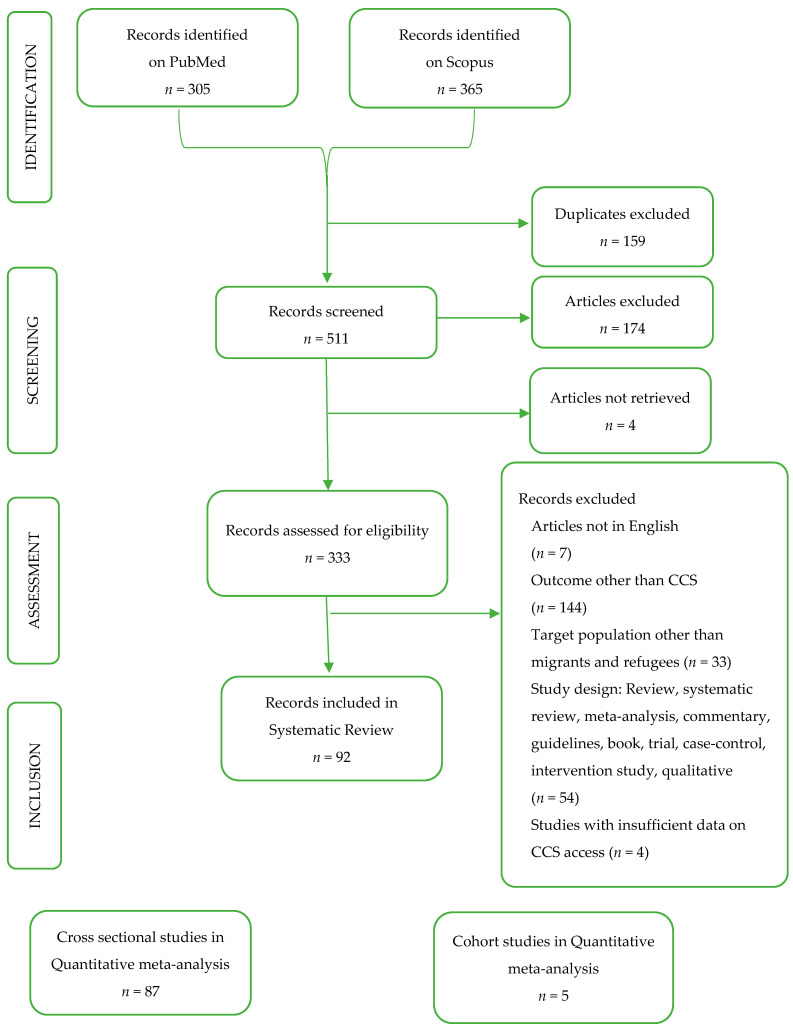
Diagram flow of the review process.

Our literature search identified 670 records, of which 305 were retrieved through the PubMed database and 365 through the Scopus database. After removing 159 duplicates, 511 reports underwent title and abstract screening, and 333 proceeded to the full-text assessment phase. The selection process, summarized in Figure 1, led to the final inclusion of 92 unique studies assessing adherence to CCS among populations with a migratory background, published between 1996 and 2024. A quantitative meta-analysis was conducted on 87 cross-sectional studies and 5 cohort studies.

Table A1, under Appendix A include the characteristics of the included studies.

Articles were evaluated as multiple different studies when they contained separate data related to either population type, income level of the country of origin, or study period. For this reason, the following analyses refer to 121 datasets (including duplicates). The included studies and their characteristics are summarized in Table 1.

### CCS Participation

A meta-analysis was conducted on 115 cross-sectional studies and 6 cohort studies that included quantitative data on screening participation.

Regular adherence to CCS was defined as having received the screening in the last 2–5 years prior the study, according to the health recommendations of the host country.

Based on the results of cross-sectional studies, the average rate of participation was 56% (95% CI: 53–60%) with a range from 10% to 93%. Refugees appeared less likely to be adherent with a rate of 49% (95% CI: 31–67%), compared to international migrants, with an adherence rate of 58% (95% CI: 55–61%). Mixed population had an adherence rate of 57% (95% CI: 51–62%).

**Figure 2:**
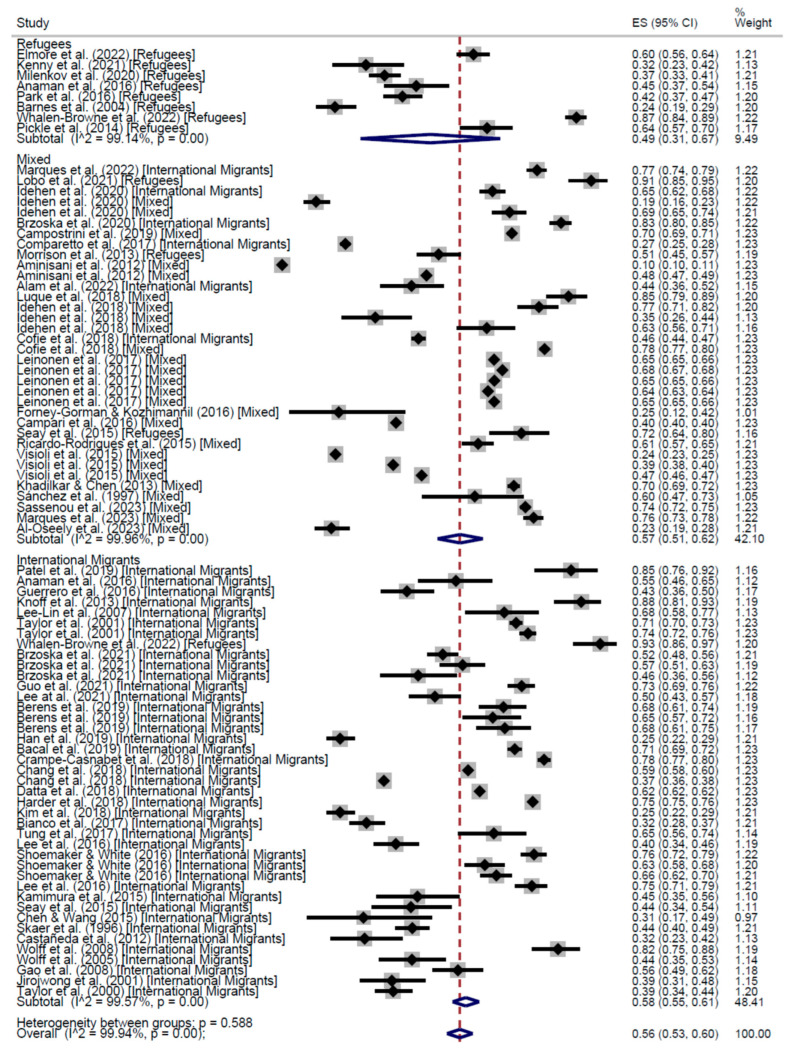
Forest plot of regular adherence to CCS by population categories, in Cross-Sectional studies [20,26,28,29,30,31,32,33,34,35,37,39,40,41,42,43,44,45,46,47,48,50,51,52,54,55,58,59,60,63,64,67,69,74,75,77,78,83,84,85,86,87,90,91,92,93,94,98,99,101,102,103,104,105,107,108,109,110,111,112].

**Figure 3:**
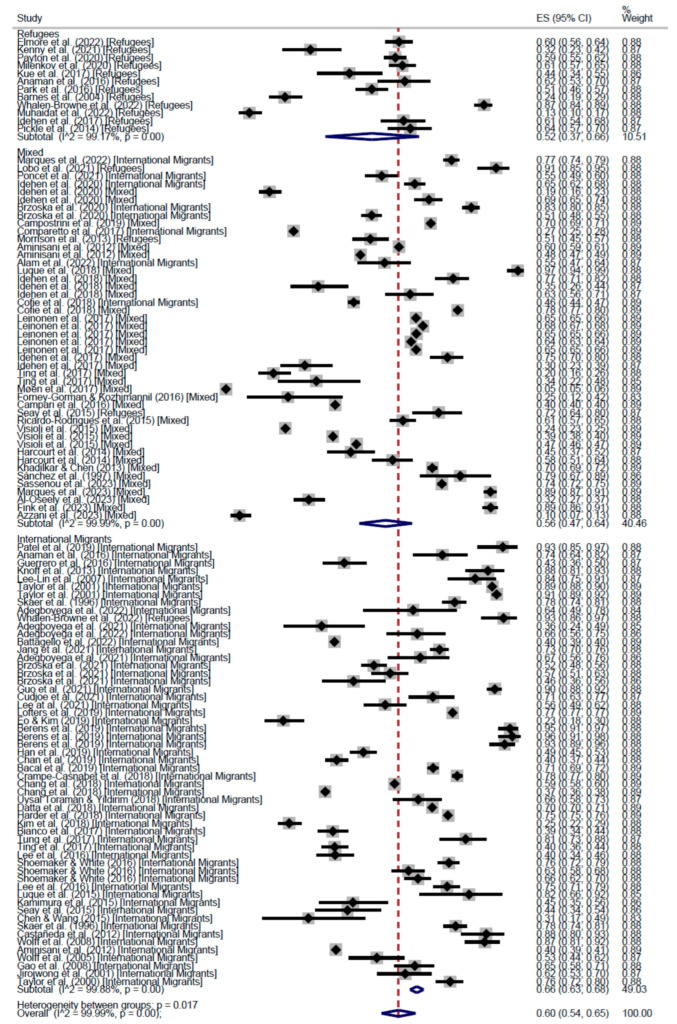
Forest plot Participation to CCS at least once, by population categories, in Cross-Sectional studies [20,26,28,29,30,31,32,33,34,35,37,39,40,41,42,43,44,45,46,47,48,50,51,52,54,55,58,59,60,63,64,67,69,73,74,75,77,78,83,84,85,86,87,90,91,92,93,94,98,99,101,102,103,104,105,107,108,109,110,111,112].

Across the 115 Cross-Sectional studies, the proportion of people who participated at least once in a CCS was 60% (95% CI: 54–65%), ranging from 0% to 97%. International migrants’ participation was 66% (95% CI: 63–68%); refugees’ participation was 52% (95% CI: 37–66%); and mixed populations participated at 56% (95% CI: 47–64%). The results are presented in Figure 2 and Figure 3.

**Figure 4:**
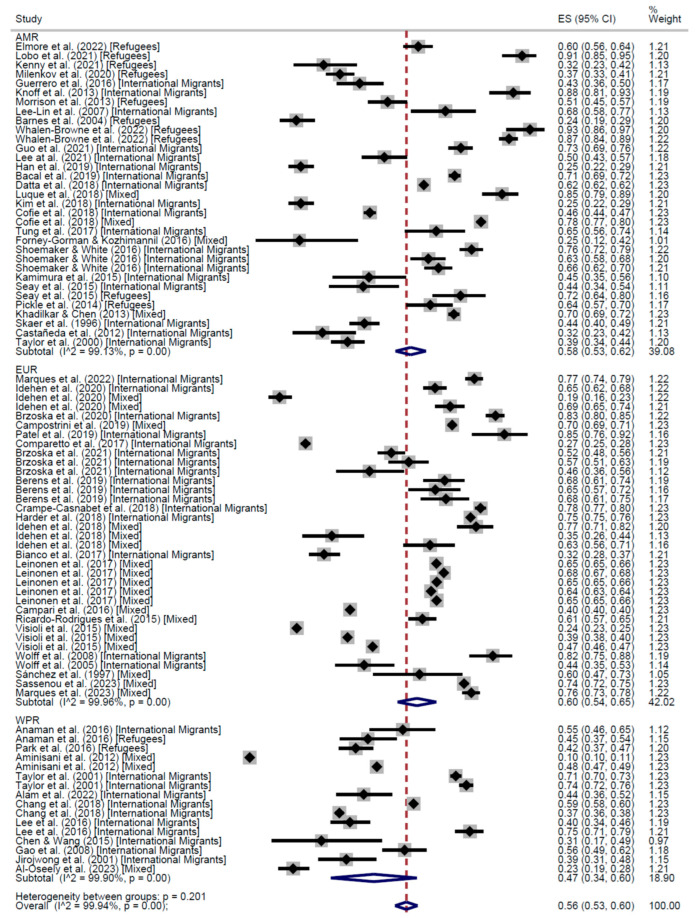
Forest plot of regular adherence to CCS, by world region, in Cross-Sectional studies [20,26,28,29,31,32,33,34,35,37,39,40,41,42,43,44,45,46,47,48,50,51,52,54,55,58,60,63,64,68,74,75,77,78,83,85,86,87,88,89,90,91,92,93,94,96,98,99,101,102,103,104,105,107,108,109,110,111,112,113].

**Figure 5:**
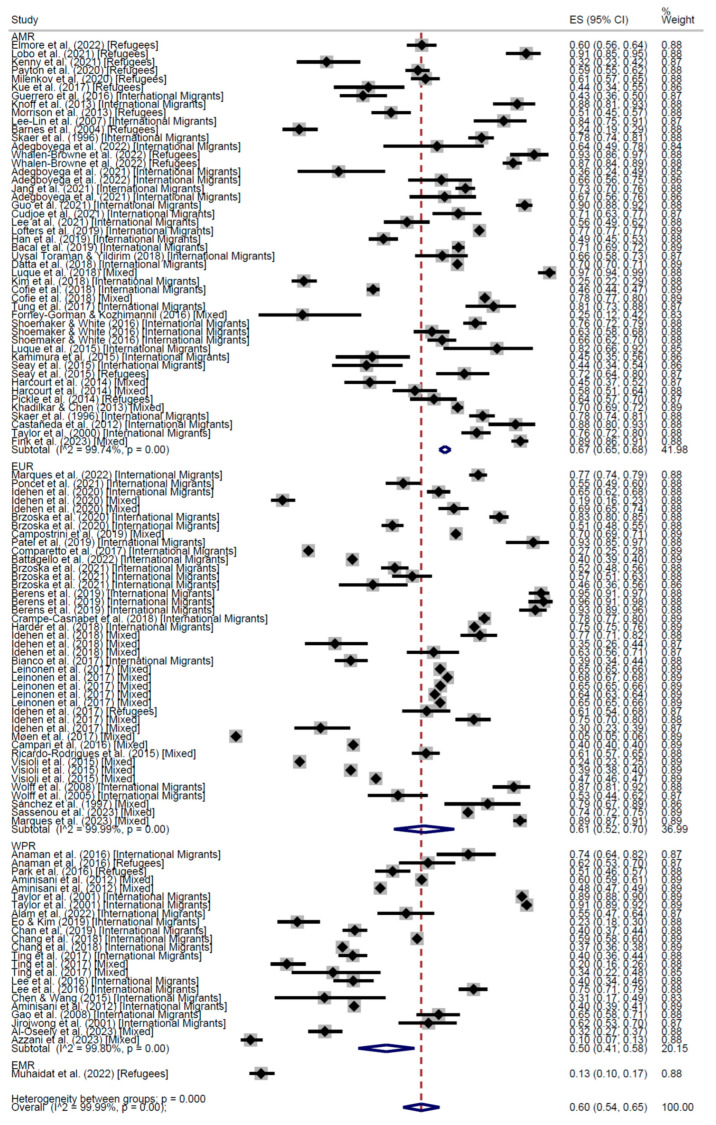
Forest plot of participation to CCS at least once, by world region, in Cross-Sectional studies [20,25,26,27,28,29,31,32,33,34,35,37,38,39,40,41,42,43,44,45,46,47,48,49,50,51,52,53,54,55,58,60,61,62,63,64,68,70,71,72,73,74,75,76,77,78,81,83,85,86,87,88,89,90,91,92,93,96,98,99,100,101,102,103,104,105,106,107,108,109,110,111,112,113,114,115].

When stratifying by world region, the results of the meta-analysis of cross-sectional studies showed regular adherence 58% in the Region of Americas (AMR) (95% CI: 53–62), 60% in the European Region (EUR) (95% CI: 54–65), and 47% in the Western Pacific Region (WPR) (95% CI: 34–60) (Figure 4). On the other hand, the proportion of people who had received the screening test at least once was higher in AMR (67%, 95% CI: 65–68) than in EUR (61%, 95% CI: 52–70), WPR (50%, 95% CI: 41–58), and the Eastern Mediterranean Region (13%, 95% CI: 10–17) (Figure 5).

**Figure 6:**
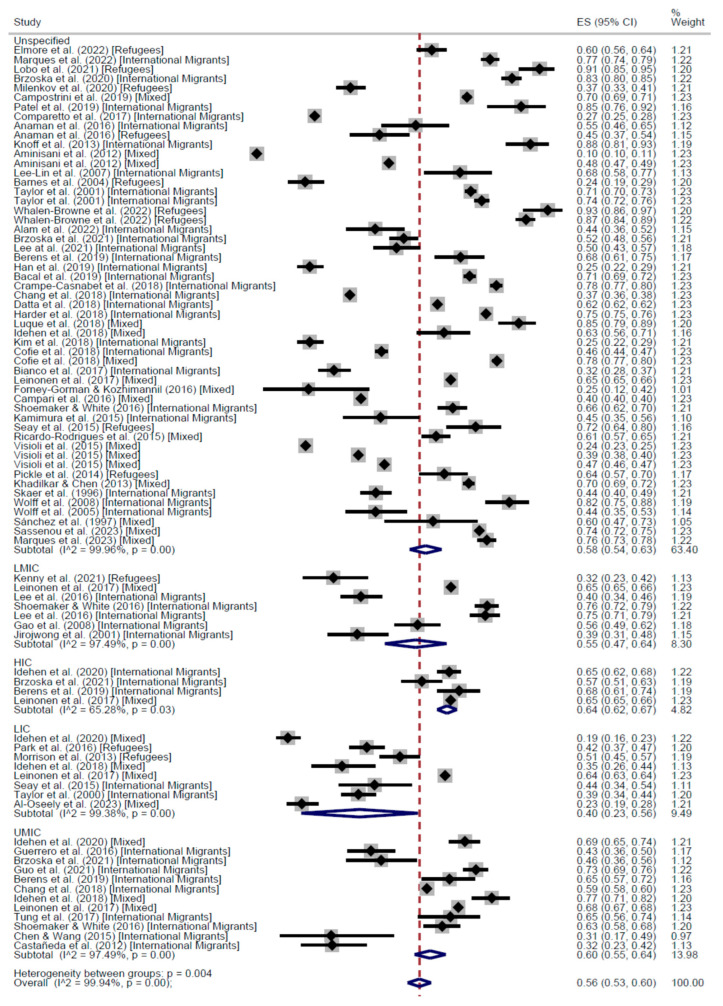
Forest plot of regular adherence to CCS, by income of the country of origin, in Cross-Sectional studies [20,26,28,29,30,31,32,33,34,35,37,39,40,41,42,44,45,46,47,48,50,51,52,54,55,58,60,63,64,67,68,74,75,77,83,84,85,86,87,90,91,92,93,94,97,98,99,101,102,103,104,105,107,108,109,110,111,112].

**Figure 7:**
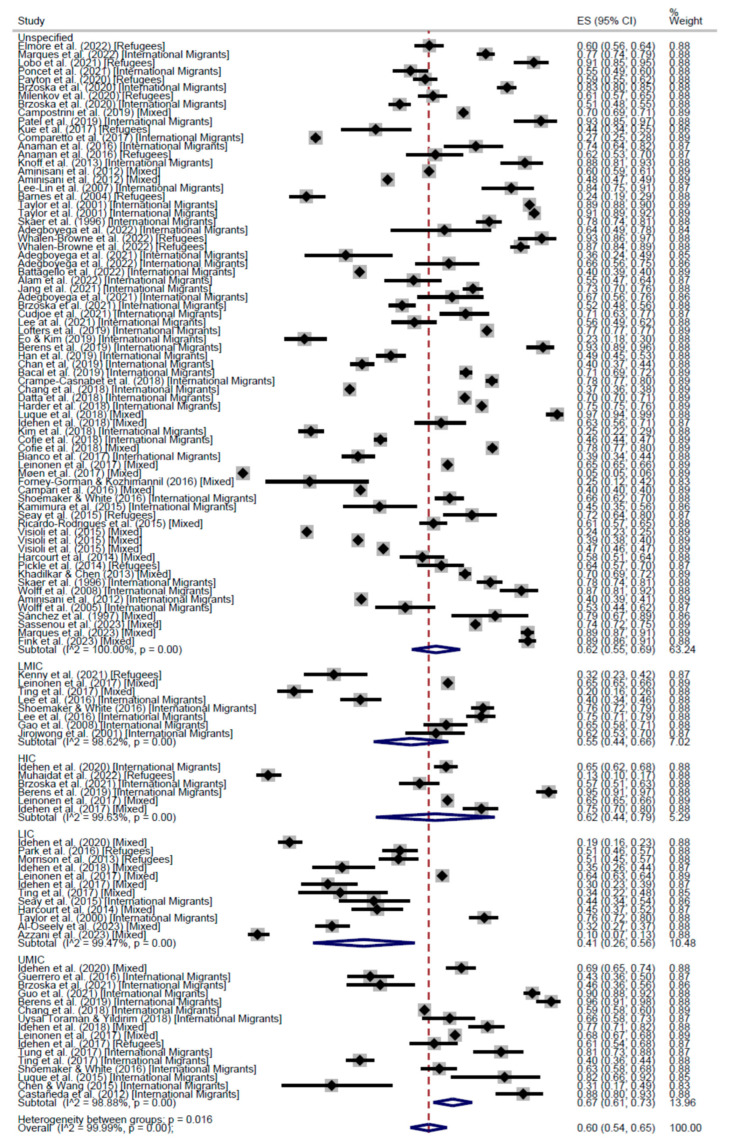
Forest plot of participation to CCS at least once, by income of the country of origin, in Cross-Sectional studies [20,26,28,29,30,31,32,33,34,35,37,38,39,40,41,42,43,44,45,46,47,48,49,50,51,52,53,54,55,58,60,63,64,67,68,74,75,77,78,83,84,85,86,87,89,90,91,92,93,94,95,96,98,99,100,101,102,103,104,105,107,108,109,110,111,112,113,114,115].

Analyzing participation by the income level of the country of origin, we grouped income levels into two main categories: High Income (including HIC and UMIC) and Low Income (including LMIC and LIC). Both regular adherence to CCS and participation at least once were higher among people from HICs compared to those from LICs and LMICs. Although, it must be noted that for most of the studies it was not possible to identify a single country of origin for the population investigated, which limited the ability to distinguish such differences clearly (Figure 6 and Figure 7).

In the meta-regression analysis of Cross-Sectional studies, adjusted for type of migrant population and study quality score, the estimated regular adherence to CSS was 57% (95% CI 50–61).

**Figure 8:**
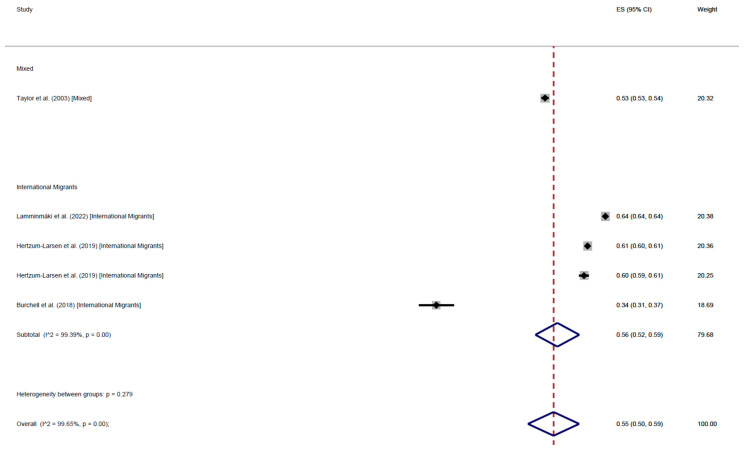
Forest plot of regular adherence to CCS, by population, in cohort studies [57,66,82,97].

**Figure 9:**
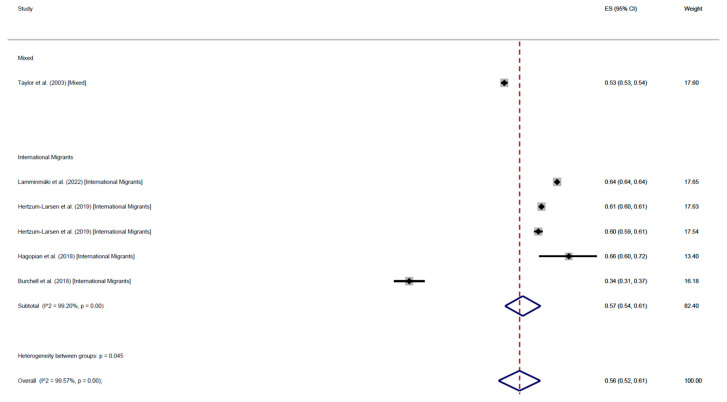
Forest plot of participation to CCS at least once, by population, in cohort studies [56,57,66,82,97].

The results of 6 cohort studies were consistent with those of cross-sectional studies: international migrants’ adherence rate was 62% (95% CI: 62–62), adherence among mixed populations was 52% (95% CI: 46–59%), with an average 55% (95% CI: 50–59%) (Figure 8). On the other hand, the mean participation at least once was 56% (95% CI: 52–61%). International migrants’ participation was 61% (95% CI: 52–70%), while participation among mixed populations was 52% (95% CI: 46–59%) (Figure 9).

All the results described above are summarized in Table 2.

The reliability of these pooled OR estimates was evaluated by examining their 95% confidence intervals, applying a random-effects model to account for between-study variation, and inspecting heterogeneity indices (I^2^). While heterogeneity was high, the consistent direction of effects across subgroups supports the robustness of the findings.

**Figure 10:**
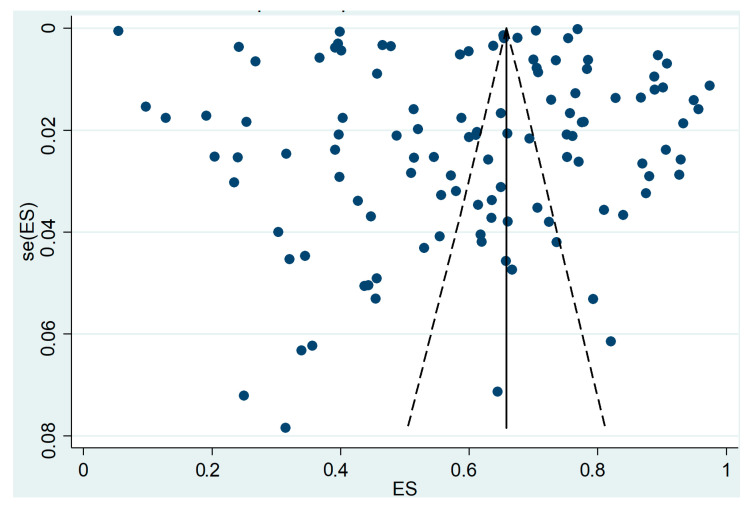
Funnel plot of cross-sectional studies.

In all the reported analyses, high between-studies heterogeneity was observed and confirmed by elevated I^2^ values. Such heterogeneity was also evident in the asymmetry of the funnel plot, which is commonly interpreted as an indicator of publication bias. Most of the cross-sectional studies were located in the upper part of the graph (Figure 10), with low standard errors, indicating more precise estimates due to large sample sizes. However, the wide horizontal scatter highlights limited comparability across studies. This likely reflects differences in study design, methodological approaches, and the influence of public health programs and migration-related determinants areas across regions.

Overall, Figure 10 indicates that while larger studies provide more precise and stable estimates, the asymmetry of the funnel plot suggests the presence of publication bias. This means that pooled estimates should be interpreted with caution, as they may be influenced by both methodological differences and selective reporting across studies.

## 4. Discussion

In this study, we critically evaluated adherence to cervical cancer screening programs (CCS). Screening of the uterine cervix, through HPV testing or cytology to identify infection or precancerous lesions, represents a key secondary prevention intervention that can substantially reduce the burden of HPV-related diseases, particularly cervical cancer, if widely implemented.

Numerous challenges exist in this field, ranging from limited epidemiological data on HPV to individual, community and structural barriers-including shortages of human and economic resources and inadequate infrastructures -that hinder effective prevention programs [116].

From the quantitative analysis, regular participation and one-time -lifetime participation was estimated at 56% and 60%, respectively, highlighting a significant healthcare need and the importance of target interventions and dedicated programs. Refugees appeared less likely to be adherent with a rate of 49% (95% CI: 31–67%), compared to international migrants. Countries of origin for refugee women may lack effective national cervical cancer control programs, hindering efforts to achieve the WHO’s call to eliminate cervical cancer by 2030 [117].

These findings directly reflect the barriers outlined in the Introduction. Lower participation rates among refugees and women from LMICs can be linked to limited awareness of the benefits of screening [89,116], reduced access to healthcare facilities and organized programs [6,118], and insufficient recommendations or outreach from healthcare providers [58]. In addition, financial difficulties and competing priorities further discourage screening, particularly among vulnerable populations [87,103,118]. Together, these obstacles provide a coherent explanation for the disparities we observed and reinforce the importance of targeted interventions to address structural and individual barriers to cervical cancer prevention.

This aligns with our finding that CCS participation rates are generally higher among individuals from high-income countries (HICs) compared to those from low- and middle-income countries (LMICs). A review by Islam [119] indicates that these settings experience major barriers to CCS, such as limited healthcare infrastructure, low awareness, and sociocultural restrictions that prevent women from seeking preventive care.

A 2020 study explored cervical cancer screening participation among migrant women in Europe, revealing that those originating from HICs were more likely to undergo screening compared to migrants from lower-income countries [120]. The disparity was attributed to several factors, including differences in healthcare infrastructure, where women from HICs had better access to organized screening programs with comprehensive coverage. Additionally, cultural perceptions played a crucial role, as women from lower-income backgrounds were more likely to encounter stigma, misinformation, or lack of awareness regarding preventive healthcare. Economic barriers further contributed to lower participation rates, as out-of-pocket costs and affordability issues often discouraged screening among these populations. A 2019 study conducted in Denmark found that immigrant women from low-income regions participated in CCS at significantly lower rates than both native Danish women and immigrants from higher-income regions. This disparity was influenced by several interrelated factors. Women from low-income countries often faced challenges in navigating the Danish healthcare system, including language barriers, limited awareness of screening programs, and difficulty accessing healthcare services. Socioeconomic constraints further contributed to lower participation, as financial hardships and competing daily priorities made preventive healthcare less of a focus [66]. 

A 2023 study by Sassenou et al. highlights that cervical cancer screening disparities are strongly influenced by the income level of a woman’s country of origin. Women from LMICs had significantly lower screening rates compared to those from wealthier nations. Limited healthcare access, financial barriers, and lower awareness of preventive care were key factors contributing to this disparity. Many women from these settings lacked familiarity with organized screening programs, and in some cases, healthcare services in their home countries were less developed, leading to lower health-seeking behaviors even after migration [111]. 

A narrative review published in 2024 [121] highlights the significant barriers and facilitators affecting cervical cancer screening uptake among migrant women across multiple countries, including the USA, UK, Canada, Australia, and the UAE. Despite the widespread availability of cervical cancer screening and vaccination programs, migrant women face considerable health disparities, leading to lower participation rates in screening services. Several barriers to screening adherence, including lack of knowledge, cultural and religious beliefs, language barriers, and socio-economic status, were identified as major obstacles. Such barriers contribute to reduced engagement with preventive healthcare and may explain the relatively low adherence rate of 58% among international migrants.

A crucial factor impacting HPV prevention is awareness and knowledge about the virus. Low participation rates in screening programs among migrants and refugees are often attributed to a lack of understanding regarding the benefits of screening [89], limited familiarity with healthcare systems, and the absence of prior screening opportunities in their countries of origin. Research indicates that migrant workers who were informed about HPV were more likely to participate in screening compared to those who had never heard of it [122].

While many women were aware of cancer, they often did not recognize its connection to HPV infection [116], underscoring the need for targeted health education programs and policies. This issue is particularly concerning because limited access to healthcare services leads many in these populations to seek medical care only in emergencies, resulting in more severe disease progression and higher healthcare costs. Greater awareness can help individuals better understand the benefits of preventive interventions and address perceived or actual risks [58].

For instance, fear of a positive screening result—and the potential diagnosis of cervical cancer—has been identified as a major barrier to participation [49], closely linked to low health literacy. This highlights a common misconception that HPV infection inevitably leads to cervical cancer, rather than recognizing that it can often be managed through preventive measures and minimally invasive treatments. Improving health literacy could significantly enhance vaccination uptake, which remains the most effective primary prevention strategy against cervical cancer [116].

Cultural factors represent another frequently cited barrier, as migrants tend to participate in screening programs at lower rates than the host population [68]. These cultural barriers can also contribute to a lack of trust in the healthcare system, particularly when addressing topics related to sexuality. For some women, the inability to access a female healthcare provider serves as a significant obstacle [41], often causing discomfort due to religious or personal beliefs.

From the language barrier perspective, another important factor is the length of residence in the host country, as a longer stay often results in improved language proficiency and a better understanding of the healthcare system [53].

To address these challenges, healthcare providers should proactively engage with individuals from migrant backgrounds by inquiring about any concerns that may affect their comfort level, thereby fostering a trusting patient-provider relationship [58]. Additionally, prevention programs specifically designed for this population, along with more culturally sensitive approaches, can significantly improve screening participation [68].

Another recurring element is the socioeconomic status. Overall, screening coverage is worse among women living in disadvantaged conditions [15]. Low income is perceived by migrant populations as a common challenge, and in countries where healthcare access depends on health insurance, being unemployed or having a low income often leads to exclusion from essential health services [103,118]. Although many countries offer free cervical cancer screening, some studies indicate that financial concerns persist within migrant populations. The perceived cost of the test itself is often seen as a barrier, despite its availability at no charge. Moreover, additional expenses, such as transportation costs to reach screening facilities or the financial burden of missing a workday, further discourage participation [87]. Consistent access to healthcare is a key predictor of the likelihood of having undergone screening. This factor is particularly significant for individuals with irregular migration status, as they often face substantial barriers to healthcare access and may be entirely excluded from preventive care systems [42].

On the other hand, social support, awareness campaigns, and the availability of screening services serve as facilitators that can improve screening uptake. Culturally sensitive approaches, such as providing education in native languages and addressing misconceptions through community-based initiatives, can help overcome these barriers. For this reason, health policies should ensure that this population is engaged as soon as possible upon arrival, e.g., at the first contact with the institutions. Greater screening participation was seen when women interacted with the health service, such as during pregnancies or hospital stays [53]. Another key factor that could help women is access to care in their own language, which facilitates asking questions and clarifying doubts about screening [65]; this applies not only to screening but also to all healthcare practices [109]. Tackling lack of knowledge and unclear communication has many positive implications for a segment of the population that now comprises more than 3% of the world population, including international migrants and refugees, almost half of whom are children [123]. 

In this context, HPV self-sampling (HPV-SST) represents a promising strategy to address many of the barriers faced by migrant and refugee women. As it allows women to collect their own sample in a private and culturally acceptable manner, self-sampling may overcome reluctance related to stigma, mistrust of the healthcare system, or limited access to female providers. Evidence from prior studies has suggested that HPV-SST can increase participation among women who are less likely to attend traditional screening programs, making it a potentially important tool to improve coverage in underserved populations [19].

Our results are consistent with and expand upon findings from recent works. For example, Islam et al. [119] and Marques et al. [120] both highlighted structural and cultural barriers as major determinants of low cervical cancer screening adherence in LMIC and migrant populations, which align with the disparities observed in our pooled analysis. Similarly, the large-scale study by Sassenou et al. [111] and the narrative review by Ozturk et al. [121] reported lower screening rates among migrant women compared to native populations, confirming the robustness of our estimates. By quantitatively synthesizing across multiple contexts, our meta-analysis provides additional support to these recent works, while also demonstrating that the magnitude of disparities remains substantial despite ongoing prevention initiatives.

Finally, there is a significant knowledge gap that hinders a comprehensive understanding of the HPV response, particularly regarding disaggregated epidemiological data, such as the estimated prevalence of cervical cancer among migrant populations. While some systematic reviews have examined this issue, research remains scarce [124,125,126]. To address this, it is crucial to integrate migrants and migration status as fundamental variables in ongoing and future HPV-related data collection, disease monitoring, research initiatives, and evaluations.

### Limitations

Our results are derived from studies conducted mainly in the Region of the Americas (mainly Canada and the USA), in the European Region (mainly Central and Western Europe) and in the Western Pacific (mainly Australia, Hong Kong, and South Korea). Therefore, data come from research conducted in HICs, which can provide more research funding, while we have limited knowledge on access to health services in LICs. Our results suggest an association between access to cervical cancer screening and the income of the country of origin. Lack of detail on this information for most of the studies made further analyses and deeper considerations difficult. However, it can be noted that lower income countries are usually the ones with less and smaller healthcare provision, especially for prevention services and therefore the ones that could benefit more from international collaboration and support. In addition, our search strategy was limited to PubMed and Scopus databases and did not include gray literature or alternative terminology such as “asylum-seekers,” “displaced persons,” or “undocumented immigrants.” While this choice ensured a focus on peer-reviewed biomedical studies, it may have excluded relevant reports from other sources. Another limitation is the restriction to English-language publications, which may have led to the exclusion of relevant studies, particularly from regions such as Europe that host large migrant and refugee populations.

Many of the studies lacked sufficient details on sociodemographic information, hindering data accuracy and the capacity to associate screening participation with migrant status. The incomplete separation between international migrants and refugees did not allow us to carry out a comprehensive analysis on this aspect.

Furthermore, many studies presented peculiar designs and investigation methods, with different population sizes and characteristics, extending the inevitable heterogeneity of the results due to the already existing differences regarding the implementation of prevention programs in various geographical regions.

Finally, it should be noted that the present study is based on observational and cross-sectional evidence synthesized through meta-analysis, rather than experimental designs. While randomized or interventional studies could provide causal insights, meta-analyses of observational studies remain a valuable approach to capture real-world adherence patterns across diverse contexts. This limitation has been acknowledged, and our findings should therefore be interpreted as indicative of associations rather than experimental proof.

A further limitation of our review is the potential presence of publication bias, as suggested by the funnel plot asymmetry (Figure 10). This may indicate that smaller or non-significant studies are underrepresented in the literature, which could influence pooled estimates. In addition, the high heterogeneity observed across studies highlights the need for cautious interpretation of our findings.

## 5. Conclusions

This meta-analysis highlights consistent inequalities and challenges in promoting effective and inclusive health policies. Prevention is now more than ever a priority for public health professionals and widening access to prevention programs can greatly reduce the disease burden, decreasing disability and premature mortality. Despite international protection policies for refugees, our data suggests that they are often excluded from access to health services. This can be attributable to the complex involvement of multiple factors involved in the migration process, and this strengthens the urge to broaden public health professionals’ perspective when implementing health promotion strategies that are tailored on refugee and migrant populations.

Inclusion also leads to significant savings in healthcare costs. Collaboration between institutions through tailored programs, perhaps in contexts where migrants first interact with government institutions and through collaborations with not-for-profit entities, could be a way to engage these populations by providing information and indications on how to move within the health system.

Furthermore, the contribution of the healthcare workforce, starting from general practitioners and pediatricians, even just by recommending preventive practices, represents a crucial facilitator. For this to be possible, however, healthcare workers need to be adequately trained to assist people with a migrant background. In 2021, the WHO produced specific guidelines and recommendations aimed at stakeholders for the implementation of training programs for healthcare personnel, who often lack the experience or skills necessary to meet the health needs of migrant or refugee people [127]. As reiterated several times, migrant and refugee may be vulnerable populations and there is a difference in the approach to the healthcare system between the migrant and the refugee populations, having the latter lived in contexts of war or coercive health systems [128]. Sometimes these people suffer from social exclusion in places where the healthcare system is inadequate [25] and they receive less assistance than the host population. While this study highlights the inequalities in access to preventive services by populations with migratory backgrounds, further studies are needed to obtain disaggregated data on determinants of access to cervical cancer screening.

In line with these findings, a simple theory of change can be outlined: barriers such as low awareness, limited access, and financial constraints reduce adherence to cervical cancer screening, while targeted interventions (e.g., culturally tailored education, patient navigation, and affordable screening options) can mitigate these obstacles. Improved adherence, in turn, supports earlier detection and contributes to achieving the WHO elimination targets. Framing the results in this way emphasizes how addressing multi-level barriers can translate directly into policy-relevant actions and measurable public health benefits.

## Figures and Tables

**Table 1:** Summary of the characteristics of the included studies [17,25,26,27,28,29,30,31,32,33,34,35,36,37,38,39,40,41,42,43,44,45,46,47,48,49,50,51,52,53,54,55,56,57,58,59,60,61,62,63,64,65,66,67,68,69,70,71,72,73,74,75,76,77,78,79,80,81,82,83,84,85,86,87,88,89,90,91,92,93,94,95,96,97,98,99,100,101,102,103,104,105,106,107,108,109,110,111,112,113,114,115].

Population	International Migrants		Refugees		Mixed			
No. of studies [Ref. number]	59 (51) * [26,27,28,29,30,31,32,33,34,35,36,37,38,39,40,41,42,43,44,45,47,48,49,50,51,52,53,54,55,56,58,60,61,62,63,64,65,71,72,73,74,75,76,77,79,80,81,82,94,97]		12 [25,41,84,86,87,88,89,90,92,95]		50 (34) * [39,46,49,57,59,66,67,68,69,70,78,83,84,89,91,96,98,99,100,101,102,103,104,105,106,107,108,109,110,111,112,113,114,115]			
World Region **	AMR		EUR		EMR		WPR	
No. of studies [Ref. number]	50 (45) * [25,26,27,28,35,37,38,39,42,44,48,50,53,54,56,57,58,59,61,64,71,72,73,75,76,77,79,80,84,85,86,88,90,91,92,93,94,99,100,103,107,108,114]		45 (28) * [31,33,46,47,51,55,60,63,66,67,68,69,70,74,78,81,82,89,96,101,102,104,105,106,109,110,111,112]		1 [111]		25 (18) * [29,30,34,36,40,41,43,45,49,52,62,65,83,87,97,98,113,115]	
	High Income			Low Income				
Origin Income	High-Income Country	Upper-Middle-Income Country		Low-Middle-Income Country		Low-Income Country		Unspecified
No. of studies [Ref. number]	8 [49,63,66,67,74,89,95,105]	16 [35,38,40,42,44,48,49,52,53,63,67,74,75,89,105,109]		8 [30,34,43,44,45,49,92,105]		13 [28,39,49,67,85,87,89,97,100,105,109,113,115]		76 (70) [20,25,26,27,29,31,32,33,36,37,39,41,44,46,47,50,51,52,54,55,56,57,58,59,60,61,62,63,64,65,66,68,69,70,71,72,73,74,77,78,79,80,81,82,83,84,86,88,90,91,93,94,96,98,99,100,101,102,103,104,105,106,107,108,109,110,111,112,114]
Study Design	Cross-Sectional	Cohort studies						
No. of studies [Ref. number]	115 (87) * [32,33,34,35,36,37,38,39,40,41,42,43,44,45,46,47,48,49,50,51,52,53,54,55,56,57,58,59,60,61,62,63,66,67,68,69,70,71,72,73,75,76,77,78,79,80,81,82,83,84,85,86,87,88,89,91,92,93,94,95,96,97,98,99,100,101,102,103,104,106,107,108,109,110,111,112,113,114,115,116,117,118,119,120,121,122,123]	6 (5) * [64,65,74,90,105]						

* Between brackets () is reported the number of unique reports for each category. ** AMR: Region of the Americas; EUR: European Region; EMR: East-Mediterranean Region; WPR: Western Pacific Region.

**Table 2:** Regular Adherence and Participation at least once to CCS by population and study type; by world region; by income of the country of origin [17,25,26,27,28,29,30,31,32,33,34,35,36,37,38,39,40,41,42,43,44,45,46,47,48,49,50,51,52,53,54,55,56,57,58,59,60,61,62,63,64,65,66,67,68,69,70,71,72,73,74,75,76,77,78,79,80,81,82,83,84,85,86,87,88,89,90,91,92,93,94,95,96,97,98,99,100,101,102,103,104,105,106,107,108,109,110,111,112,113,114,115]. (IM: International Migrants. R: Refugees. Mix: Mixed. AMR: Region of the Americas. EUR: European Region. WPR: Western Pacific Region. EMR: Eastern Mediterranean Region).

	Adherence				Participation at Least Once				
	IM	R	Mix	Total	IM	R	Mix		Total
Cross-Sectional	57% (95% CI: 53–61)	55% (95% CI: 40–70)	55% (95% CI: 48–62)	56% (95% CI: 53–60)	66% (95% CI: 63–68)	52% (95% CI: 37–66)	56% (95% CI: 47–64)		60% (95% CI: 54–65)
Cohort	62% (95% CI: 62–62)	-	52% (95% CI: 46–59)	55% (95% CI: 50–59)	61% (95% CI: 52–70)	-	52% (95% CI: 46–59)		56% (95% CI: 52–61)
	**AMR**	**EUR**	**WPR**	**Total**	**AMR**	**EUR**	**WPR**	**EMR**	**Total**
Cross-Sectional	58% (95% CI: 53–62)	60% (95% CI: 54–65)	47% (95% CI: 34–60)	56% (95% CI: 53–60)	67% (95% CI: 65–68)	61% (95% CI: 52–70)	50% (95% CI: 41–58)	13% (95% CI: 10–17)	60% (95% CI: 54–65)
	**High income**	**Low income**	**Unspecified**	**Total**	**High income**	**Low** **income**	**Unspecified**		**Total**
Cross-Sectional	63% (95% CI: 60–65)	47% (95% CI: 42–52)	58% (95% CI: 54–63)	56% (95% CI: 53–60)	66% (95% CI: 62–69)	47% (95% CI: 41–52)	62% (95% CI: 55–69)		60% (95% CI: 54–65)

## Data Availability

No new data were created or analyzed in this study. Data sharing is not applicable to this article.

## Appendix A

**Table A1:** Characteristics of the included studies (year) [with reference no.].

Author (Year)	Population (N)	Region	Economic Level	Study Design	QS
Payton et al. (2020) [25]	Refugees (782)	AMR	Unspecified	Cross-Sectional	6
Skaer et al. (1996) [26]	International Migrants (512)	AMR	Unspecified	Cross-Sectional	4
Skaer et al. (1996) [27]	International Migrants (510)	AMR	Unspecified	Cross-Sectional	7
Taylor et al. (2000) [28]	International Migrants (406)	AMR	LIC	Cross-Sectional	6
Taylor et al. (2001) [29]	International Migrants (3392)	WPR	Unspecified	Cross-Sectional	7
Taylor et al. (2001) [29]	International Migrants (1743)	WPR	Unspecified	Cross-Sectional	7
Jirojwong et al. (2001) [30]	International Migrants (134)	WPR	LMIC	Cross-Sectional	4
Wolff et al. (2005) [31]	International Migrants (134)	EUR	Unspecified	Cross-Sectional	1
Lee-Lin et al. (2007) [32]	International Migrants (100)	AMR	Unspecified	Cross-Sectional	7
Wolff et al. (2008) [33]	International Migrants (161)	EUR	Unspecified	Cross-Sectional	4
Gao et al. (2008) [34]	International Migrants (234)	WPR	LMIC	Cross-Sectional	8
Castañeda et al. (2012) [35]	International Migrants (104)	AMR	UMIC	Cross-Sectional	2
Aminisani et al. (2012) [36]	International Migrants (12,392)	WPR	Unspecified	Cross-Sectional	9
Knoff et al. (2013) [37]	International Migrants (125)	AMR	Unspecified	Cross-Sectional	5
Luque et al. (2015) [38]	International Migrants (39)	AMR	UMIC	Cross-Sectional	3
Kamimura et al. (2015) [17]	International Migrants (88)	AMR	Unspecified	Cross-Sectional	2
Seay et al. (2015) [39]	International Migrants (96)	AMR	LIC	Cross-Sectional	3
Seay et al. (2015) [39]	Unspecified (138)	AMR	Unspecified	Cross-Sectional	3
Chen & Wang (2015) [40]	International Migrants (35)	WPR	UMIC	Cross-Sectional	3
Anaman et al. (2016) [41]	International Migrants (110)	WPR	Unspecified	Cross-Sectional	6
Anaman et al. (2016) [41]	Refugees (144)	WPR	Unspecified	Cross-Sectional	6
Guerrero et al. (2016) [42]	International Migrants (213)	AMR	UMIC	Cross-Sectional	4
Lee et al. (2016) [43]	International Migrants (281)	WPR	LMIC	Cross-Sectional	4
Shoemaker & White (2016) [44]	International Migrants (659)	AMR	LMIC	Cross-Sectional	6
Shoemaker & White (2016) [44]	International Migrants (351)	AMR	UMIC	Cross-Sectional	6
Shoemaker & White (2016) [44]	International Migrants (526)	AMR	Unspecified	Cross-Sectional	6
Lee et al. (2016) [45]	International Migrants (427)	WPR	LMIC	Cross-Sectional	6
Comparetto et al. (2017) [46]	Unspecified (4609)	EUR	Unspecified	Cross-Sectional	6
Bianco et al. (2017) [47]	International Migrants (419)	EUR	Unspecified	Cross-Sectional	5
Tung et al. (2017) [48]	International Migrants (121)	AMR	UMIC	Cross-Sectional	4
Ting et al. (2017) [49]	International Migrants (549)	WPR	UMIC	Cross-Sectional	4
Ting et al. (2017) [49]	Unspecified (255)	WPR	LMIC	Cross-Sectional	4
Ting et al. (2017) [49]	Unspecified (5)	WPR	HIC	Cross-Sectional	4
Ting et al. (2017) [49]	Unspecified (56)	WPR	LIC	Cross-Sectional	4
Bacal et al. (2019) [50]	International Migrants (2782)	AMR	Unspecified	Cross-Sectional	8
Crampe-Casnabet et al. (2018) [51]	International Migrants (2637)	EUR	Unspecified	Cross-Sectional	5
Chang et al. (2018) [52]	International Migrants (9067)	WPR	UMIC	Cross-Sectional	9
Chang et al. (2018) [52]	International Migrants (6868)	WPR	Unspecified	Cross-Sectional	9
Uysal Toraman & Yildirim (2018) [53]	International Migrants (156)	AMR	UMIC	Cross-Sectional	5
Datta et al. (2018) [54]	International Migrants (826,387)	AMR	Unspecified	Cross-Sectional	6
Harder et al. (2018) [55]	International Migrants (48,218)	EUR	Unspecified	Cross-Sectional	8
Hagopian et al. (2018) [56]	International Migrants (271)	AMR	Unspecified	Cohort	4
Burchell et al. (2018) [57]	Unspecified (949)	AMR	Unspecified	Cohort	4
Kim et al. (2018) [58]	International Migrants (560)	AMR	Unspecified	Cross-Sectional	5
Cofie et al. (2018) [59]	Unspecified (3080)	AMR	Unspecified	Cross-Sectional	5
Patel et al. (2019) [60]	International Migrants (82)	EUR	Unspecified	Cross-Sectional	5
Lofters et al. (2019) [61]	International Migrants (3,630,981)	AMR	Unspecified	Cross-Sectional	9
Eo & Kim (2019) [62]	International Migrants (196)	WPR	Unspecified	Cross-Sectional	5
Berens et al. (2019) [63]	International Migrants (239)	EUR	HIC	Cross-Sectional	3
Berens et al. (2019) [63]	International Migrants (163)	EUR	UMIC	Cross-Sectional	3
Berens et al. (2019) [63]	International Migrants (179)	EUR	Unspecified	Cross-Sectional	3
Han et al. (2019) [64]	International Migrants (560)	AMR	Unspecified	Cross-Sectional	4
Chan et al., (2019) [65]	International Migrants (776)	WPR	Unspecified	Cross-Sectional	5
Hertzum-Larsen et al. (2019) [66]	Unspecified (12,500)	EUR	HIC	Cohort	8
Hertzum-Larsen et al. (2019) [66]	Unspecified (44,829)	EUR	Unspecified	Cohort	8
Idehen et al. (2020) [67]	Unspecified (816)	EUR	HIC	Cross-Sectional	9
Idehen et al. (2020) [67]	Unspecified (523)	EUR	LIC	Cross-Sectional	9
Idehen et al. (2020) [67]	Unspecified (451)	EUR	UMIC	Cross-Sectional	9
Brzoska et al. (2020) [68]	Unspecified (755)	EUR	Unspecified	Cross-Sectional	7
Brzoska et al. (2020) [69]	Unspecified (983)	EUR	Unspecified	Cross-Sectional	8
Poncet et al. (2021) [70]	Unspecified (387)	EUR	Unspecified	Cross-Sectional	5
Adegboyega et al. (2021) [71]	International Migrants (59)	AMR	Unspecified	Cross-Sectional	5
Jang et al. (2021) [72]	International Migrants (999)	AMR	Unspecified	Cross-Sectional	6
Adegboyega et al. (2021) [73]	International Migrants (99)	AMR	Unspecified	Cross-Sectional	6
Brzoska et al. (2021) [74]	International Migrants (292)	EUR	HIC	Cross-Sectional	6
Brzoska et al. (2021) [74]	International Migrants (103)	EUR	UMIC	Cross-Sectional	6
Brzoska et al. (2021) [74]	International Migrants (634)	EUR	Unspecified	Cross-Sectional	6
Guo et al. (2021) [75]	International Migrants (652)	AMR	UMIC	Cross-Sectional	4
Cudjoe et al. (2021) [76]	International Migrants (167)	AMR	Unspecified	Cross-Sectional	5
Lee at al. (2021) [77]	International Migrants (230)	AMR	Unspecified	Cross-Sectional	4
Marques et al. (2022) [78]	Unspecified (1100)	EUR	Unspecified	Cross-Sectional	7
Adegboyega et al. (2022) [79]	International Migrants (45)	AMR	Unspecified	Cross-Sectional	5
Adegboyega et al. (2022) [80]	International Migrants (108)	AMR	Unspecified	Cross-Sectional	4
Battagello et al. (2022) [81]	International Migrants (26,355)	EUR	Unspecified	Cross-Sectional	8
Lamminmäki et al. (2022) [82]	International Migrants (85,272)	EUR	Unspecified	Cohort	8
Alam et al. (2022) [83]	Unspecified (148)	WPR	Unspecified	Cross-Sectional	5
Barnes et al. (2004) [84]	Refugees (283)	AMR	Unspecified	Cross-Sectional	5
Morrison et al. (2013) [85]	Unspecified (310)	AMR	LIC	Cross-Sectional	7
Pickle et al. (2014) [86]	Refugees (203)	AMR	Unspecified	Cross-Sectional	5
Park et al. (2016) [87]	Refugees (385)	WPR	LIC	Cross-Sectional	5
Kue et al. (2017) [88]	Refugees (97)	AMR	Unspecified	Cross-Sectional	5
Idehen et al. (2017) [89]	Refugees (197)	EUR	UMIC	Cross-Sectional	6
Idehen et al. (2017) [89]	Unspecified (291)	EUR	HIC	Cross-Sectional	6
Idehen et al. (2017) [89]	Unspecified (132)	EUR	LIC	Cross-Sectional	6
Milenkov et al. (2020) [90]	Refugees (542)	AMR	Unspecified	Cross-Sectional	6
Lobo et al. (2021) [91]	Unspecified (149)	AMR	Unspecified	Cross-Sectional	5
Kenny et al. (2021) [92]	Refugees (106)	AMR	LMIC	Cross-Sectional	5
Elmore et al. (2022) [93]	Refugees (525)	AMR	Unspecified	Cross-Sectional	10
Whalen-Browne et al. (2022) [94]	International Migrants (99)	AMR	Unspecified	Cross-Sectional	8
Muhaidat et al. (2022) [95]	Refugees (359)	EMR	HIC	Cross-Sectional	4
Sánchez et al. (1997) [96]	Unspecified (58)	EUR	Unspecified	Cross-Sectional	7
Taylor et al. (2003) [97]	International Migrants (22,787)	WPR	LIC	Cohort	5
Aminisani et al. (2012) [98]	Unspecified (11,477)	WPR	Unspecified	Cross-Sectional	8
Aminisani et al. (2012) [98]	Unspecified (19,907)	WPR	Unspecified	Cross-Sectional	8
Khadilkar & Chen (2013) [99]	Unspecified (3420)	AMR	Unspecified	Cross-Sectional	7
Harcourt et al. (2014) [100]	Unspecified (181)	AMR	LIC	Cross-Sectional	4
Harcourt et al. (2014) [100]	Unspecified (238)	AMR	Unspecified	Cross-Sectional	4
Ricardo-Rodrigues et al. (2015) [101]	Unspecified (570)	EUR	Unspecified	Cross-Sectional	6
Visioli et al. (2015) [102]	Unspecified (13,535)	EUR	Unspecified	Cross-Sectional	8
Visioli et al. (2015) [102]	Unspecified (16,427)	EUR	Unspecified	Cross-Sectional	8
Visioli et al. (2015) [102]	Unspecified (22,319)	EUR	Unspecified	Cross-Sectional	8
Forney-Gorman & Kozhimannil (2016) [103]	Unspecified (36)	AMR	Unspecified	Cross-Sectional	7
Campari et al. (2016) [104]	Unspecified (516,291)	EUR	Unspecified	Cross-Sectional	6
Leinonen et al. (2017) [105]	Unspecified (112,801)	EUR	HIC	Cross-Sectional	9
Leinonen et al. (2017) [105]	Unspecified (58,644)	EUR	UMIC	Cross-Sectional	9
Leinonen et al. (2017) [105]	Unspecified (58,888)	EUR	LMIC	Cross-Sectional	9
Leinonen et al. (2017) [105]	Unspecified (19,260)	EUR	LIC	Cross-Sectional	9
Leinonen et al. (2017) [105]	Unspecified (67,999)	EUR	Unspecified	Cross-Sectional	9
Møen et al. (2017) [106]	Unspecified (152,800)	EUR	Unspecified	Cross-Sectional	8
Cofie et al. (2018) [107]	Unspecified (4278)	AMR	Unspecified	Cross-Sectional	8
Luque et al. (2018) [108]	Unspecified (196)	AMR	Unspecified	Cross-Sectional	6
Idehen et al. (2018) [109]	Unspecified (167)	EUR	Unspecified	Cross-Sectional	7
Idehen et al. (2018) [109]	Unspecified (257)	EUR	UMIC	Cross-Sectional	7
Idehen et al. (2018) [109]	Unspecified (113)	EUR	LIC	Cross-Sectional	7
Campostrini et al. (2019) [110]	Unspecified (5576)	EUR	Unspecified	Cross-Sectional	7
Sassenou et al. (2023) [111]	Unspecified (4891)	EUR	Unspecified	Cross-Sectional	5
Marques et al. (2023) [112]	Unspecified (1100)	EUR	Unspecified	Cross-Sectional	7
Al-Oseely et al. (2023) [113]	Unspecified (355)	WPR	LIC	Cross-Sectional	6
Fink et al. (2023) [114]	Unspecified (675)	AMR	Unspecified	Cross-Sectional	4
Azzani et al. (2023) [115]	Unspecified (370)	WPR	LIC	Cross-Sectional	6

## References

[1] Khan I., Harshithkumar R., More A., Mukherjee A. (2023). Human Papilloma Virus: An Unraveled Enigma of Universal Burden of Malignancies. Pathogens.

[2] Montgomery A., Durden A., Sundararajan S., Al-Booz H., Newton C. (2023). Review of invasive cervical cancer. Obstet. Gynaecol. Reprod. Med..

[3] Seyoum A., Assefa N., Gure T., Seyoum B., Mulu A., Mihret A. (2022). Prevalence and Genotype Distribution of High-Risk Human Papillomavirus Infection Among Sub-Saharan African Women: A Systematic Review and Meta-Analysis. Front. Public Health.

[4] Bruni L., Albero G., Serrano B., Mena M., Collado J.J., Gómez D., Muñoz J., Bosch F.X., de Sanjosé S., ICO/IARC Information Centre on HPV and Cancer (HPV Information Centre) Human Papillomavirus and Related Diseases in the World. Summary Report 10 March 2023. https://hpvcentre.net/statistics/reports/XWX.pdf.

[5] Ardekani A., Sepidarkish M., Mollalo A., Afradiasbagharani P., Rouholamin S., Rezaeinejad M., Farid-Mojtahedi M., Mahjour S., Almukhtar M., Nourollahpour Shiadeh M. (2023). Worldwide prevalence of human papillomavirus among pregnant women: A systematic review and meta-analysis. Rev. Med. Virol..

[6] Zhetpisbayeva I., Kassymbekova F., Sarmuldayeva S., Semenova Y., Glushkova N. (2023). Cervical Cancer Prevention in Rural Areas. Ann. Glob. Health.

[7] Sung H., Ferlay J., Siegel R.L., Laversanne M., Soerjomataram I., Jemal A., Bray F. (2021). Global Cancer Statistics 2020: GLOBOCAN Estimates of Incidence and Mortality Worldwide for 36 Cancers in 185 Countries. CA Cancer J. Clin..

[8] Drolet M., Bénard E., Boily M.-C., Ali H., Baandrup L., Bauer H., Beddows S., Brisson J., Brotherton J.M.L., Cummings T. (2015). Population-level impact and herd effects following human papillomavirus vaccination programmes: A systematic review and meta-analysis. Lancet Infect. Dis..

[9] Castle P.E., Kinney W.K., Cheung L.C., Gage J.C., Fetterman B., Poitras N.E., Lorey T.S., Wentzensen N., Befano B., Schussler J. (2017). Why does cervical cancer occur in a state-of-the-art screening program?. Gynecol. Oncol..

[10] Choi S., Ismail A., Pappas-Gogos G., Boussios S. (2023). HPV and Cervical Cancer: A Review of Epidemiology and Screening Uptake in the UK. Pathogens.

[11] WHO (2024). Cervical Cancer. World Health Organization Website. https://www.who.int/news-room/fact-sheets/detail/cervical-cancer?gclid=CjwKCAiAk9itBhASEiwA1my_69sUl9sTor9BNLOUnMPxJFd1RbaSs_I4-9Z3mfyvs1BAOfQFMkOwFxoCLtwQAvD_BwE.

[12] Jain M., Yadav D., Jarouliya U., Chavda V., Yadav A.K., Chaurasia B., Song M. (2023). Epidemiology, Molecular Pathogenesis, Immuno-Pathogenesis, Immune Escape Mechanisms and Vaccine Evaluation for HPV-Associated Carcinogenesis. Pathogens.

[13] Elemes S., Stachteas P., Haidich A.B., Mamopoulos A., Smyrnakis E. (2023). The Impact of the COVID-19 Pandemic on Breast and Cervical Cancer Screening: A Systematic Review. In Vivo.

[14] Swanson A.A., Pantanowitz L. (2024). The evolution of cervical cancer screening. J. Am. Soc. Cytopathol..

[15] Waller J., McCaffery K., Forrest S., Szarewski A., Cadman L., Austin J., Wardle J. (2006). Acceptability of unsupervised HPV self-sampling using written instructions. J. Med. Screen..

[16] Strelow B., O’Laughlin D. (2022). Barriers to cervical cancer screening among immigrants. JAAPA.

[17] Kamimura A., Myers K., Ashby J., Trinh H.N., Nourian M.M., Reel J.J. (2015). Women in Free Clinics: An Assessment of Health-Related Quality of Life for Prevention and Health Education. J. Community Health.

[18] Ponce-Chazarri L., Ponce-Blandón J.A., Immordino P., Giordano A., Morales F. (2023). Barriers to Breast Cancer-Screening Adherence in Vulnerable Populations. Cancers.

[19] Marshall S., Vahabi M., Lofters A. (2019). Acceptability, Feasibility and Uptake of HPV Self-Sampling Among Immigrant Minority Women: A Focused Literature Review. J. Immigr. Minor. Health.

[20] Page M.J., McKenzie J.E., Bossuyt P.M., Boutron I., Hoffmann T.C., Mulrow C.D., Shamseer L., Tetzlaff J.M., Akl E.A., Moher D. (2021). The PRISMA 2020 statement: An updated guideline for reporting systematic reviews. J. Clin. Epidemiol..

[21] Convention Relating to the Status of Refugees (1951). United Nations Conference of Plenipotentiaries on the Status of Refugees and Stateless Persons. https://www.ohchr.org/sites/default/files/refugees.pdf.

[22] WHO Organizational Structure. https://www.who.int/about/structure.

[23] The World Bank World Development Indicators—Historical Classification by Income. https://datacatalog.worldbank.org/search/dataset/0037712.

[24] Wells G.A., Shea B., O’Connell D., Peterson J., Welch V., Losos M., Tugwell P. The Newcastle-Ottawa Scale (NOS) for Assessing the Quality of Nonrandomised Studies in Meta-Analyses. https://www.ohri.ca/programs/clinical_epidemiology/oxford.asp.

[25] Payton C., Zeidan A., Bogen H., Altshuler M. (2020). Women’s Health Screening and Mapped Community Resources for Refugees Resettled in Philadelphia, Pennsylvania. J. Health Care Poor Underserved.

[26] Skaer T.L., Robison L.M., Sclar D.A., Harding G.H. (1996). Knowledge, attitudes, and patterns of cancer screening: A self-report among foreign born Hispanic women utilizing rural migrant health clinics. J. Rural Health.

[27] Skaer T.L., Robison L.M., Sclar D.A., Harding G.H. (1996). Cancer-screening determinants among Hispanic women using migrant health clinics. J. Health Care Poor Underserved.

[28] Taylor V.M., Jackson J.C., Yasui Y., Schwartz S.M., Kuniyuki A., Fischer M., Tu S.P. (2000). Pap Testing Stages of Adoption among Cambodian Immigrants. Asian Am. Pac. Isl. J. Health.

[29] Taylor R.J., Mamoon H.A., Morrell S.L., Wain G.V. (2001). Cervical screening in migrants to Australia. Aust. N. Z. J. Public Health.

[30] Jirojwong S., Maclennan R., Manderson L. (2001). Health beliefs and Pap smears among Thai women in Brisbane, Australia. Asia-Pac. J. Public Health.

[31] Wolff H., Stalder H., Epiney M., Walder A., Irion O., Morabia A. (2005). Health care and illegality: A survey of undocumented pregnant immigrants in Geneva. Soc. Sci. Med. (1982).

[32] Lee-Lin F., Pett M., Menon U., Lee S., Nail L., Mooney K., Itano J. (2007). Cervical cancer beliefs and pap test screening practices among Chinese American immigrants. Oncol. Nurs. Forum.

[33] Wolff H., Epiney M., Lourenco A.P., Costanza M.C., Delieutraz-Marchand J., Andreoli N., Dubuisson J.B., Gaspoz J.M., Irion O. (2008). Undocumented migrants lack access to pregnancy care and prevention. BMC Public Health.

[34] Gao W., Paterson J., DeSouza R., Lu T. (2008). Demographic predictors of cervical cancer screening in Chinese women in New Zealand. N. Z. Med. J..

[35] Castañeda S.F., Rosenbaum R.P., Gonzalez P., Holscher J.T. (2012). Breast and cervical cancer screening among rural midwestern latina migrant and seasonal farmworkers. J. Prim. Care Community Health.

[36] Aminisani N., Armstrong B.K., Canfell K. (2012). Participation in cervical screening by older asian and middle eastern migrants in new South wales, australia. Health Promot. Perspect..

[37] Knoff J.S., Harlow S.D., Yassine M., Soliman A.S. (2013). Cervical cancer screening practice and knowledge among Hispanic migrant and seasonal farmworkers of Michigan. J. Prim. Care Community Health.

[38] Luque J.S., Tarasenko Y.N., Maupin J.N., Alfonso M.L., Watson L.C., Reyes-Garcia C., Ferris D.G. (2015). Cultural beliefs and understandings of cervical cancer among Mexican immigrant women in Southeast Georgia. J. Immigr. Minor. Health.

[39] Seay J.S., Carrasquillo O., Campos N.G., McCann S., Amofah A., Pierre L., Kobetz E. (2015). Cancer Screening Utilization Among Immigrant Women in Miami, Florida. Prog. Community Health Partnersh..

[40] Chen W.T. (2013). Chinese female immigrants english-speaking ability and breast and cervical cancer early detection practices in the New York metropolitan area. Asian Pac. J. Cancer Prev..

[41] Anaman J.A., Correa-Velez I., King J. (2017). A survey of cervical screening among refugee and non-refugee African immigrant women in Brisbane, Australia. Health Promot. J. Aust..

[42] Guerrero N., Zhang X., Rangel G., Gonzalez-Fagoaga J.E., Martinez-Donate A. (2016). Cervical and Breast Cancer Screening Among Mexican Migrant Women, 2013. Prev. Chronic Dis..

[43] Lee F.H., Wang H.H., Yang Y.M., Huang J.J., Tsai H.M. (2016). Influencing Factors of Intention to Receive Pap Tests in Vietnamese Women who Immigrated to Taiwan for Marriage. Asian Nurs. Res..

[44] Shoemaker M.L., White M.C. (2016). Breast and cervical cancer screening among Asian subgroups in the USA: Estimates from the National Health Interview Survey, 2008, 2010, and 2013. Cancer Causes Control.

[45] Lee F.H., Wang H.H., Tsai H.M., Lin M.L. (2016). Factors associated with receiving Pap tests among married immigrant women of Vietnamese origin in southern Taiwan. Women Health.

[46] Comparetto C., Epifani C., Manca M.C., Lachheb A., Bravi S., Cipriani F., Bellomo F., Olivieri S., Fiaschi C., Di Marco L. (2017). Uptake of cervical cancer screening among the migrant population of Prato Province, Italy. Int. J. Gynaecol. Obstet..

[47] Bianco A., Larosa E., Pileggi C., Nobile C.G.A., Pavia M. (2017). Cervical and breast cancer screening participation and utilisation of maternal health services: A cross-sectional study among immigrant women in Southern Italy. BMJ Open.

[48] Tung W.C., Lu M., Granner M. (2017). Perceived Benefits and Barriers of Cervical Cancer Screening Among Chinese American Women. Oncol. Nurs. Forum.

[49] Ting Y.H., Tse H.Y., Lam W.C., Chan K.S., Leung T.Y. (2017). The pattern of cervical smear abnormalities in marginalised women in Hong Kong. Hong Kong Med. J. Xianggang Yi Xue Za Zhi.

[50] Bacal V., Blinder H., Momoli F., Wu K.Y., McFaul S. (2019). Is Immigrant Status Associated with Cervical Cancer Screening Among Women in Canada? Results From a Cross-Sectional Study. J. Obstet. Gynaecol. Can..

[51] Crampe-Casnabet C., Franck J.E., Ringa V., Coeuret-Pellicer M., Chauvin P., Menvielle G. (2018). Role of obesity in differences in cervical cancer screening rates by migration history. The CONSTANCES survey. Cancer Epidemiol..

[52] Chang H.K., Seo S.S., Myong J.P., Koo J.W., Jeong J. (2018). Factors Associated with Cervical Cancer Screening among Married Female Immigrants with Korean Husbands in South Korea. Int. J. Environ. Res. Public Health.

[53] Uysal Toraman A., Yildirim N. (2018). Knowledge About Cervical Cancer Risk Factors and Practices of Pap Testing Among Turkish Immigrant Women in the United States. J. Immigr. Minor. Health.

[54] Datta G.D., Blair A., Sylvestre M.P., Gauvin L., Drouin M., Mayrand M.H. (2018). Cervical cancer screening in Montreal: Building evidence to support primary care and policy interventions. Prev. Med..

[55] Harder E., Juul K.E., Jensen S.M., Thomsen L.T., Frederiksen K., Kjaer S.K. (2018). Factors associated with non-participation in cervical cancer screening—A nationwide study of nearly half a million women in Denmark. Prev. Med..

[56] Hagopian G.S., Lieber M., Dottino P.R., Margaret Kemeny M., Li X., Overbey J., Clark L.D., Beddoe A.M. (2018). The impact of nativity on cervical cancer survival in the public hospital system of Queens, New York. Gynecol. Oncol..

[57] Burchell A.N., Kendall C.E., Cheng S.Y., Lofters A., Cotterchio M., Bayoumi A.M., Glazier R.H., Antoniou T., Raboud J., Yudin M.H. (2018). Cervical cancer screening uptake among HIV-positive women in Ontario, Canada: A population-based retrospective cohort study. Prev. Med..

[58] Kim K., Xue Q.L., Walton-Moss B., Nolan M.T., Han H.R. (2018). Decisional balance and self-efficacy mediate the association among provider advice, health literacy and cervical cancer screening. Eur. J. Oncol. Nurs..

[59] Cofie L.E., Hirth J.M., Guo F., Berenson A.B., Markides K., Wong R. (2018). HPV Vaccination Among Foreign-Born Women: Examining the National Health Interview Survey 2013–2015. Am. J. Prev. Med..

[60] Patel H., Sherman S.M., Tincello D., Moss E.L. (2020). Awareness of and attitudes towards cervical cancer prevention among migrant Eastern European women in England. J. Med. Screen..

[61] Lofters A.K., Kopp A., Vahabi M., Glazier R.H. (2019). Understanding those overdue for cancer screening by five years or more: A retrospective cohort study in Ontario, Canada. Prev. Med..

[62] Eo Y.S., Kim J.S. (2019). Associations of health belief and health literacy with Pap smear practice among Asian immigrant women. Eur. J. Oncol. Nurs..

[63] Berens E.M., Mohwinkel L.M., van Eckert S., Reder M., Kolip P., Spallek J. (2019). Uptake of Gynecological Cancer Screening and Performance of Breast Self-Examination Among 50-Year-Old Migrant and Non-migrant Women in Germany: Results of a Cross-Sectional Study (InEMa). J. Immigr. Minor. Health.

[64] Han H.R., Kim K., Cudjoe J., Kim M.T. (2019). Familiarity, Navigation, and Comprehension: Key Dimensions of Health Literacy in Pap Test Use among Korean American Women. J. Health Commun..

[65] Chan D.N.S., So W.K.W., Choi K.C., Gurung S. (2019). Development of an explanatory model to explore cervical cancer screening behaviour among South Asian women: The influence of multilevel factors. Eur. J. Oncol. Nurs..

[66] Hertzum-Larsen R., Kjær S.K., Frederiksen K., Thomsen L.T. (2019). Participation in cervical cancer screening among immigrants and Danish-born women in Denmark. Prev. Med..

[67] Idehen E.E., Virtanen A., Lilja E., Tuomainen T.P., Korhonen T., Koponen P. (2020). Cervical Cancer Screening Participation among Women of Russian, Somali, and Kurdish Origin Compared with the General Finnish Population: A Register-Based Study. Int. J. Environ. Res. Public Health.

[68] Brzoska P., Aksakal T., Yilmaz-Aslan Y. (2020). Disparities in the use of regular pap smears among migrant and non-migrant women in Austria: A population-based survey of 7633 women. J. Med. Screen..

[69] Brzoska P., Aksakal T., Yilmaz-Aslan Y. (2020). Utilization of cervical cancer screening among migrants and non-migrants in Germany: Results from a large-scale population survey. BMC Public Health.

[70] Poncet L., Panjo H., Ringa V., Andro A. (2021). Do vulnerable groups access prevention services? Cervical cancer screening and HIV testing among homeless migrant women in the Paris metropolitan area. PLoS ONE.

[71] Adegboyega A., Wiggins A.T., Williams L.B., Dignan M. (2021). HPV Testing Behaviors and Willingness to Use HPV Self-sampling at Home Among African American (AA) and Sub-Saharan African Immigrant (SAI) Women. J. Racial Ethn. Health Disparities.

[72] Jang S.H., Meischke H., Ko L.K. (2021). The impact of medical tourism on cervical cancer screening among immigrant women in the U.S. BMC Women’s Health.

[73] Adegboyega A., Wu J.R., Mudd-Martin G. (2021). Acculturation Strategies and Pap Screening Uptake among Sub-Saharan African Immigrants (SAIs). Int. J. Environ. Res. Public Health.

[74] Brzoska P., Wahidie D., Yilmaz-Aslan Y. (2021). An Intersectional Perspective on the Utilization of Cervical Cancer Screening among Migrants. A Cross-Sectional Analysis of Survey Data from Austria. Cancers.

[75] Guo X.M., Tom L., Leung I., O’Brian C., Zumpf K., Simon M. (2021). Associations between Fatalistic Cancer Beliefs and Cancer-Screening Behaviors in Chinese American Immigrant Women. J. Immigr. Minor. Health.

[76] Cudjoe J., Budhathoki C., Roter D., Gallo J.J., Sharps P., Han H.R. (2021). Exploring Health Literacy and the Correlates of Pap Testing Among African Immigrant Women: Findings from the AfroPap Study. J. Cancer Educ..

[77] Lee H.Y., Choi Y.J., Shin J., Yoon Y.J., An S. (2021). Adherence to Cervical Cancer Screening in Korean American Immigrant Women: Identifying Malleable Variables for Intervention Development. J. Transcult. Nurs..

[78] Marques P., Geraldes M., Gama A., Heleno B., Dias S. (2022). Non-attendance in cervical cancer screening among migrant women in Portugal: A cross-sectional study. Women’s Health.

[79] Adegboyega A., Wiggins A.T., Obielodan O., Dignan M., Schoenberg N. (2022). Beliefs associated with cancer screening behaviors among African Americans and Sub-Saharan African immigrant adults: A cross-sectional study. BMC Public Health.

[80] Adegboyega A., Aroh A., Williams L.B., Mudd-Martin G. (2022). Social support and cervical cancer screening among sub-Saharan African immigrant (SAI) women. Cancer Causes Control.

[81] Battagello J., Monetti D., Rizzato S., Rosano A., Stocco C.F., Zamberlan S., Rugge M., Zorzi M. (2022). Young immigrant women and cervical cancer screening: Participation and lesions detected at the first screening round. Epidemiol. Prev..

[82] Lamminmäki M., Leivonen A., Sarkeala T., Virtanen A., Heinävaara S. (2022). Health inequalities among Russian-born immigrant women in Finland: Longitudinal analysis on cervical cancer incidence and participation in screening. J. Migr. Health.

[83] Alam Z., Ann Dean J., Janda M. (2022). Cervical screening uptake: A cross-sectional study of self-reported screening attitudes, behaviours and barriers to participation among South Asian immigrant women living in Australia. Women’s Health.

[84] Barnes D.M., Harrison C.L. (2004). Refugee women’s reproductive health in early resettlement. J. Obstet. Gynecol. Neonatal Nurs..

[85] Morrison T.B., Flynn P.M., Weaver A.L., Wieland M.L. (2013). Cervical cancer screening adherence among Somali immigrants and refugees to the United States. Health Care Women Int..

[86] Pickle S., Altshuler M., Scott K.C. (2014). Cervical Cancer Screening Outcomes in a Refugee Population. J. Immigr. Refug. Stud..

[87] Park J., Kim H., Yang W., Lee H., Park S.M. (2018). Cervical Cancer Screening and Its Associated Factors Among North Korean Defectors Living in South Korea. J. Immigr. Minor. Health.

[88] Kue J., Hanegan H., Tan A. (2017). Perceptions of Cervical Cancer Screening, Screening Behavior, and Post-Migration Living Difficulties Among Bhutanese-Nepali Refugee Women in the United States. J. Community Health.

[89] Idehen E.E., Korhonen T., Castaneda A., Juntunen T., Kangasniemi M., Pietilä A.M., Koponen P. (2017). Factors associated with cervical cancer screening participation among immigrants of Russian, Somali and Kurdish origin: A population-based study in Finland. BMC Women’s Health.

[90] Milenkov A.R., Felini M., Baker E., Acharya R., Longanga Diese E., Onsa S., Fernando S., Chor H. (2020). Uptake of cancer screenings among a multiethnic refugee population in North Texas, 2014–2018. PLoS ONE.

[91] Lobo S.J., Lin J.G., Vais S., Wang D., Adegoke T.M., Wu W.J., Steer-Massaro C. (2021). Pap Smear and Mammogram Screening Rates in a Refugee and General OB/GYN Clinic: A Retrospective Review. J. Immigr. Minor. Health.

[92] Kenny D.X., Hsueh K., Walters R.W., Coté J.J. (2021). Human Papillomavirus Vaccination and Pap Smear Rates Among Burmese Refugee Girls in a Healthcare System in Omaha, Nebraska. J. Community Health.

[93] Elmore C.E., Mitchell E.M., Debnam K., Keim-Malpass J., Laughon K., Tanabe K.O., Hauck F.R. (2022). Predictors of cervical cancer screening for refugee women attending an international family medicine clinic in the United States. Cancer Causes Control.

[94] Whalen-Browne M., Talavlikar R., Brown G., McBrien K., Wiedmeyer M.L., Norrie E., Fabreau G. (2022). Cervical Cancer Screening by Refugee Category in a Refugee Health Primary Care Clinic in Calgary, Canada, 2011–2016. J. Immigr. Minor. Health.

[95] Muhaidat N., Alshrouf M.A., Alshajrawi R.N., Miqdadi Z.R., Amro R., Rabab’ah A.O., Qatawneh S.A., Albandi A.M., Fram K. (2022). Cervical Cancer Screening among Female Refugees in Jordan: A Cross-Sectional Study. Healthcare.

[96] Sánchez V., Rohlfs I., Borràs J.M., Borrell C. (1997). Migration within Spain, level of education, and cervical cancer screening. Eur. J. Cancer Prev..

[97] Taylor R.J., Morrell S.L., Mamoon H.A., Macansh S., Ross J., Wain G.V. (2003). Cervical cancer screening in a Vietnamese nominal cohort. Ethn. Health.

[98] Aminisani N., Armstrong B.K., Canfell K. (2012). Cervical cancer screening in Middle Eastern and Asian migrants to Australia: A record linkage study. Cancer Epidemiol..

[99] Khadilkar A., Chen Y. (2013). Rate of cervical cancer screening associated with immigration status and number of years since immigration in Ontario, Canada. J. Immigr. Minor. Health.

[100] Harcourt N., Ghebre R.G., Whembolua G.L., Zhang Y., Warfa Osman S., Okuyemi K.S. (2014). Factors associated with breast and cervical cancer screening behavior among African immigrant women in Minnesota. J. Immigr. Minor. Health.

[101] Ricardo-Rodrigues I., Jiménez-García R., Hernández-Barrera V., Carrasco-Garrido P., Jiménez-Trujillo I., López de Andrés A. (2015). Social disparities in access to breast and cervical cancer screening by women living in Spain. Public Health.

[102] Visioli C.B., Crocetti E., Zappa M., Iossa A., Andersson K.L., Bulgaresi P., Alfieri A., Amunni G. (2015). Participation and risk of high grade cytological lesions among immigrants and Italian-born women in an organized cervical cancer screening program in Central Italy. J. Immigr. Minor. Health.

[103] Forney-Gorman A., Kozhimannil K.B. (2016). Differences in Cervical Cancer Screening Between African-American Versus African-Born Black Women in the United States. J. Immigr. Minor. Health.

[104] Campari C., Fedato C., Iossa A., Petrelli A., Zorzi M., Anghinoni E., Bietta C., Brachini A., Brezzi S., Cogo C. (2016). Cervical cancer screening in immigrant women in Italy: A survey on participation, cytology and histology results. Eur. J. Cancer Prev..

[105] Leinonen M.K., Campbell S., Ursin G., Tropé A., Nygård M. (2017). Barriers to cervical cancer screening faced by immigrants: A registry-based study of 1.4 million women in Norway. Eur. J. Public Health.

[106] Møen K.A., Kumar B., Qureshi S., Diaz E. (2017). Differences in cervical cancer screening between immigrants and nonimmigrants in Norway: A primary healthcare register-based study. Eur. J. Cancer Prev..

[107] Cofie L.E., Hirth J.M., Wong R. (2018). Chronic comorbidities and cervical cancer screening and adherence among US-born and foreign-born women. Cancer Causes Control.

[108] Luque J.S., Tarasenko Y.N., Li H., Davila C.B., Knight R.N., Alcantar R.E. (2018). Utilization of Cervical Cancer Screening Among Hispanic Immigrant Women in Coastal South Carolina. J. Racial Ethn. Health Disparities.

[109] Idehen E.E., Koponen P., Härkänen T., Kangasniemi M., Pietilä A.M., Korhonen T. (2018). Disparities in cervical screening participation: A comparison of Russian, Somali and Kurdish immigrants with the general Finnish population. Int. J. Equity Health.

[110] Campostrini S., Carrozzi G., Severoni S., Masocco M., Salmaso S., WHO Migration Health Programme, Office of the Regional Director, WHO Regional Office for Europe, PASSI National Coordinating Group (2019). Migrant health in Italy: A better health status difficult to maintain-country of origin and assimilation effects studied from the Italian risk factor surveillance data. Popul. Health Metr..

[111] Sassenou J., Ringa V., Zins M., Ozguler A., Paquet S., Panjo H., Franck J.E., Menvielle G., Rigal L. (2023). Combined influence of immigration status and income on cervical cancer screening uptake. Prev. Med. Rep..

[112] Marques P., Geraldes M., Gama A., Heleno B., Dias S. (2023). What is the role of attitudinal barriers on cervical cancer screening non-attendance? Findings from a cross-sectional study with migrant women in Portugal. BMC Women’s Health.

[113] Al-Oseely S., Abdul Manaf R., Ismail S. (2023). Factors affecting cervical cancer screening among Yemeni immigrant women in Klang Valley, Malaysia: A Cross-Sectional study. PLoS ONE.

[114] Fink G., Abdulcadir J., Johnson-Agbakwu C.E. (2023). Rates of Cervical Cancer Screening and Dysplasia Among Refugees in a Health Care Safety Net System. J. Immigr. Minor. Health.

[115] Azzani M., Ba-Alawi E., Atroosh W.M., Yadav H. (2023). Awareness of cervical cancer and its associated socio-demographic factors among Yemeni immigrant women in Malaysia. BMC Women’s Health.

[116] Bhatta M.P., Johnson D.C., Lama M., Maharjan B., Lhaki P., Shrestha S. (2020). Cervical Cancer and Human Papillomavirus Vaccine Awareness Among Married Bhutanese Refugee and Nepali Women in Eastern Nepal. J. Community Health.

[117] Elmore C.E., Keim-Malpass J., Mitchell E.M. (2021). Health Inequity in Cervical Cancer Control Among Refugee Women in the United States by Country of Origin. Health Equity.

[118] Miranda P.Y., Yao N., Snipes S.A., BeLue R., Lengerich E., Hillemeier M.M. (2017). Citizenship, length of stay, and screening for breast, cervical, and colorectal cancer in women, 2000–2010. Cancer Causes Control.

[119] Islam R.M., Billah B., Hossain M.N., Oldroyd J. (2017). Barriers to Cervical Cancer and Breast Cancer Screening Uptake in Low-Income and Middle-Income Countries: A Systematic Review. Asian Pac. J. Cancer Prev..

[120] Marques P., Nunes M., Antunes M.D.L., Heleno B., Dias S. (2020). Factors associated with cervical cancer screening participation among migrant women in Europe: A scoping review. Int. J. Equity Health.

[121] Ozturk N.Y., Hossain S.Z., Mackey M., Adam S., Brennan P. (2024). HPV and Cervical Cancer Awareness and Screening Practices among Migrant Women: A Narrative Review. Healthcare.

[122] Holt H.K., Zhang X., Hu S.Y., Zhao F.H., Smith J.S., Qiao Y.L. (2021). Inequalities in Cervical Cancer Screening Uptake Between Chinese Migrant Women and Local Women: A Cross-Sectional Study. Cancer Control J. Moffitt Cancer Cent..

[123] Dossier Statistico Immigrazione 2023 Italia, Europa e le Nuove Politiche Migratorie. Dati e Riflessioni Nel Nuovo Rapporto a Cura Del Centro Studi e Ricerche Idos. https://integrazionemigranti.gov.it/it-it/Ricerca-news/Dettaglio-news/id/3478/Dossier-statistico-immigrazione-023#:~:text=A%20fine%202022%20si%20stimano,essi%20%C3%A8%20costituito%20da%20minorenn.

[124] Graci D., Piazza N., Ardagna S., Casuccio A., Drobov A., Geraci F., Immordino A., Pirrello A., Restivo V., Rumbo R. (2024). Barriers to and Facilitators for Accessing HPV Vaccination in Migrant and Refugee Populations: A Systematic Review. Vaccines.

[125] Essa-Hadad J., Gorelik Y., Vervoort J., Jansen D., Edelstein M. (2024). Understanding the health system barriers and enablers to childhood MMR and HPV vaccination among disadvantaged, minority or underserved populations in middle- and high-income countries: A systematic review. Eur. J. Public Health.

[126] Rosato I., Dalla Zuanna T., Tricarico V., Barbiellini Amidei C., Canova C. (2023). Adherence to Cervical Cancer Screening Programs in Migrant Populations: A Systematic Review and Meta-Analysis. Int. J. Environ. Res. Public Health.

[127] World Health Organization (2021). Refugee and Migrant Health: Global Competency Standards for Health Workers (The Standards). https://www.who.int/publications/i/item/9789240030626.

[128] Ghebrendrias S., Pfeil S., Crouthamel B., Chalmiers M., Kully G., Mody S. (2021). An Examination of Misconceptions and Their Impact on Cervical Cancer Prevention Practices among Sub-Saharan African and Middle Eastern Refugees. Health Equity.

